# Drivers of Decline in Diarrhea Mortality Between GEMS and VIDA Studies

**DOI:** 10.1093/cid/ciad015

**Published:** 2023-04-19

**Authors:** Emily L Deichsel, Helen Powell, Christopher Troeger, M Jahangir Hossain, Samba O Sow, Richard Omore, Momodou Jasseh, Uma Onwuchekwa, David Obor, Doh Sanogo, Joquina Chiquita M Jones, Dilruba Nasrin, Milagritos D Tapia, Karen L Kotloff

**Affiliations:** Center for Vaccine Development and Global Health, University of Maryland School of Medicine, Baltimore, Maryland, USA; Department of Pediatrics, University of Maryland School of Medicine, Baltimore, Maryland, USA; Center for Vaccine Development and Global Health, University of Maryland School of Medicine, Baltimore, Maryland, USA; Department of Pediatrics, University of Maryland School of Medicine, Baltimore, Maryland, USA; Institute for Health Metrics and Evaluation, University of Washington, Seattle, Washington, USA; Medical Research Council Unit The Gambia at London School of Hygiene and Tropical Medicine, Banjul, The Gambia; Centre pour le Développement des Vaccins du Mali (CVD-Mali), Bamako, Mali; Kenya Medical Research Institute, Center for Global Health Research (KEMRI-CGHR), Kisumu, Kenya; Medical Research Council Unit The Gambia at London School of Hygiene and Tropical Medicine, Banjul, The Gambia; Centre pour le Développement des Vaccins du Mali (CVD-Mali), Bamako, Mali; Kenya Medical Research Institute, Center for Global Health Research (KEMRI-CGHR), Kisumu, Kenya; Centre pour le Développement des Vaccins du Mali (CVD-Mali), Bamako, Mali; Medical Research Council Unit The Gambia at London School of Hygiene and Tropical Medicine, Banjul, The Gambia; Center for Vaccine Development and Global Health, University of Maryland School of Medicine, Baltimore, Maryland, USA; Department of Medicine, University of Maryland School of Medicine, Baltimore, Maryland, USA; Center for Vaccine Development and Global Health, University of Maryland School of Medicine, Baltimore, Maryland, USA; Department of Pediatrics, University of Maryland School of Medicine, Baltimore, Maryland, USA; Center for Vaccine Development and Global Health, University of Maryland School of Medicine, Baltimore, Maryland, USA; Department of Pediatrics, University of Maryland School of Medicine, Baltimore, Maryland, USA; Department of Medicine, University of Maryland School of Medicine, Baltimore, Maryland, USA

**Keywords:** GEMS, VIDA, rotavirus vaccine

## Abstract

**Background:**

Statistical modeling suggests that decreasing diarrhea-associated mortality rates in recent decades are largely attributed to improved case management, rotavirus vaccine, and economic development.

**Methods:**

We examined data collected in 2 multisite population-based diarrhea case-control studies, both conducted in The Gambia, Kenya, and Mali: the Global Enteric Multicenter Study (GEMS; 2008–2011) and Vaccine Impact on Diarrhea in Africa (VIDA; 2015–2018). Population-level diarrhea mortality and risk factor prevalence, estimated using these study data, were used to calculate the attribution of risk factors and interventions for diarrhea mortality using a counterfactual framework. We performed a decomposition of the effects of the changes in exposure to each risk factor between GEMS and VIDA on diarrhea mortality for each site.

**Results:**

Diarrhea mortality among children under 5 in our African sites decreased by 65.3% (95% confidence interval [CI]: –80.0%, −45.0%) from GEMS to VIDA. Kenya and Mali had large relative declines in diarrhea mortality between the 2 periods with 85.9% (95% CI: −95.1%, −71.5%) and 78.0% (95% CI: −96.0%, 36.3%) reductions, respectively. Among the risk factors considered, the largest declines in diarrhea mortality between the 2 study periods were attributed to reduction in childhood wasting (27.2%; 95% CI: −39.3%, −16.8%) and an increased rotavirus vaccine coverage (23.1%; 95% CI: −28.4%, −19.4%), zinc for diarrhea treatment (12.1%; 95% CI: −16.0%, −8.9%), and oral rehydration salts (ORS) for diarrhea treatment (10.2%).

**Conclusions:**

The VIDA study sites demonstrated exceptional reduction in diarrhea mortality over the last decade. Site-specific differences highlight an opportunity for implementation science in collaboration with policymakers to improve the equitable coverage of these interventions globally.

## Teaser

We demonstrated exceptional reduction in diarrhea mortality over a decade. Among the risk factors considered, the largest declines in diarrhea mortality were attributed to reduction in childhood wasting and an increased rotavirus vaccine coverage, zinc, and oral rehydration salts for diarrhea treatment.

The mortality from diarrhea has decreased among children in recent decades, in part due to successful implementation of effective programs and interventions to prevent and treat diarrhea in high-burden areas [1, 2]. However, diarrhea remains a leading cause of mortality among children younger than 5 years old, with sub-Saharan Africa bearing the highest burden [3]. Identifying drivers of diarrhea mortality over time will aid policymakers in determining resource allocation in order to accelerate and maintain recent declines, which will be critical to achieve 2020 Sustainable Development Goals of eliminating preventable childhood deaths.

Statistical modeling suggests that global decreases in diarrhea-associated mortality rates in recent decades are largely attributed to improved case management, rotavirus vaccine implementation, and economic development [4, 5]. Few studies have sufficient data to assess drivers using data collected directly for a local setting. The availability of primary data collected on diarrhea mortality and risk factors will complement statistical models in order to understand the contributors to of the decline in diarrhea mortality.

The Global Enteric Multicenter Study (GEMS) was a prospective, matched case-control study of the burden of moderate-to-severe diarrhea (MSD) among children (2008–2011) in 7 countries in sub-Saharan Africa and South Asia [6]. Three of the 7 country sites from GEMS participated in a follow-on study, Vaccine Impact on Diarrhea in Africa (VIDA), with the goal of assessing the etiology and burden of MSD in children under 5 years following introduction of rotavirus vaccine using similar methodology (2015–2018). Herein we report our observations of a decline in diarrhea mortality over the 10-year period spanning these 2 studies in the 3 country sites—The Gambia, Kenya, and Mali—concomitant with changes in social, environmental, and economic conditions, in addition to introduction and high coverage rates for rotavirus vaccine. GEMS and VIDA operated under comparable protocols, providing a large data resource and a unique opportunity to examine changes in the prevalence of risk factors and interventions for diarrhea mortality over time and the impact these changes may have had in the decline in diarrhea mortality in these study sites. We also report the current mortality rates and risk factor contributions to mortality as seen in VIDA.

## METHODS

### Data Collection

The study design and clinical methods used in GEMS and VIDA have been described previously [6, 7]. In brief, this analysis is restricted to the 3 sites (with minor geographic modifications) that participated in both GEMS and VIDA: Basse and Bansang (VIDA only), The Gambia; Siaya County, Kenya; and Bamako, Mali. Each country site provided a censused population with an ongoing demographic surveillance system (DSS) updated 2 to 4 times per year, and supplemented with weekly reports of intercurrent birth and death events by local informants. We used the median population from DSS rounds for each age and site stratum as the study population sizes in all analyses. The DSS rounds were accompanied by a Healthcare Utilization Survey (HUS) among a randomly selected, age-stratified sample of children in the DSS to collect healthcare-seeking information for recent diarrheal episodes.

Children aged 0–59 months were included if they were a DSS resident who presented to a sentinel health facility over the 36-month study period with new (onset after 7 diarrhea-free days) and acute (onset within the previous 7 days) episode of MSD, defined as 1 or more of the following: dehydration (sunken eyes, loss of skin turgor, or intravenous rehydration required), dysentery, or recommended admission to the hospital. Enrollments were age stratified: infants (0–11 months), toddlers (12–23 months), and children (24–59 months). One to 3 randomly selected controls without MSD were matched to each case by age, sex, and neighborhood.

Cases and controls provided clinical and epidemiological information using structured parental interviews; questions were largely uniform across the 2 studies, with unsafe sanitation being 1 exception (differences between the questions are detailed in Table 1). Cases and controls were followed up at home approximately 60 (range 50–90 days) days after enrollment to assess vital status. Follow-up visits were completed for 91% of cases in GEMS and 96% of cases in VIDA. For each site and age stratum, the proportion of children identified in the HUS as having an incident case of MSD in the previous week and whose parents reported that they sought care at a study sentinel hospital or health center within 7 days of diarrhea onset (*r* value) was used to derive incident diarrhea mortality estimates for the entire DSS (see Blackwelder et al [14] for more detail).

**Table 1.** Definition and Relative Risk for Risk Factors Associated With Diarrhea Mortality Used in Analysis

Risk Factor	Relative Risk	Definition	Ref
Vaccination status with rotavirus vaccine (among controls)			[8]
ȃNot fully vaccinated	1.0^a^	Not receiving all rotavirus vaccine doses independent of age (<3 doses in Mali [RotaTeq, pentavalent, Merck Vaccines, Whitehouse Station, New Jersey]), <2 doses in Kenya (Rotarix, monovalent, GlaxoSmithKline Biologics, Rixenstart, Belgium), <3 doses in The Gambia (RotaTeq) if first dose occurred on or before 3 April 2017, <2 doses (Rotarix) in The Gambia if first dose occurred after 3 April 2017. All participants at all sites in GEMS were considered not fully vaccinated.	
ȃFully vaccinated	0.69	Received all rotavirus vaccine doses independent of age (3 doses in Mali [RotaTeq], 2 doses in Kenya [Rotarix], 3 doses in The Gambia [RotaTeq] if first dose occurred on or before 3 April 2017, 2 doses in The Gambia [Rotarix] if first dose occurred after 3 April 2017).	
Unsafe sanitation (among controls)			[4, 9]
ȃSafely managed	1.0^a^	GEMS: No shared flush toilet, no shared ventilated improved pit latrine, no shared pour-flush toilet, no shared ventilated improved pit latrine with water seal VIDA: No shared flush or pour-flush toilet to piped, sewer system septic tank, pit latrine, no shared flush or pour-flush toilet to elsewhere, no shared ventilated improved pit latrine	
ȃImproved	2.60	GEMS: Shared flush toilet, shared ventilated improved pit latrine, shared pour-flush toilet, shared ventilated improved pit latrine with water seal VIDA: Shared flush or pour-flush toilet to piped, sewer system septic tank, pit latrine, shared flush or pour-flush toilet to elsewhere, shared ventilated improved pit latrine	
ȃUnimproved/no facility	3.242	GEMS: Traditional pit toilet, no facility, ventilated improved pit VIDA: Bucket, pit latrine with slap pit latrine without slab or open pit, composting toilet, hanging toilet or hanging latrine, no facility	
Unsafe water (among controls)			[4, 9]
ȃPiped	3.511^a^	Piped into house	
ȃImproved	3.926	Public tap, deep tube well, shallow tube well, covered well in house or yard, covered public well, protected spring, rainwater, bought (tank/bottles), bore hole	
ȃUnimproved	4.789	Open well in house or yard, open public well, pond or lake, unprotected spring, river, dam or earth, stream	
Childhood stunting (among controls)			[4, 10]
ȃNone	1.0^a^	HAZ ≥ −1.0	
ȃMild	1.11	−2.0 ≤ HAZ < −1.0	
ȃModerate	1.22	−3.0 ≤ HAZ < −2.0	
ȃSevere	1.85	HAZ < −3.0	
Childhood underweight (among controls)			[4, 10]
ȃNone	1.0^a^	WAZ ≥ −1.0	
ȃMild	1.09	−2.0 ≤ WAZ < −1.0	
ȃModerate	1.23	−3.0 ≤ WAZ < −2.0	
ȃSevere	2.33	WAZ < −3.0	
Childhood wasting (among controls)			[4, 10]
ȃNone	1.0^a^	WHZ ≥ −1.0	
ȃMild	6.60	−2.0 ≤ WHZ < −1.0	
ȃModerate	23.26	−3.0 ≤ WHZ < −2.0	
ȃSevere	105.76	WHZ < −3.0	
No ORS for treatment (among cases)			[11]
ȃReceived ORS or home fluids for treatment	1.0^a^	Administered ORS or extra fluids at home or in health facility	
ȃNo ORS or home fluids for treatment	3.23	Not administered ORS or extra fluids at home nor in health facility	
No zinc for treatment (among cases)			[5, 12]
ȃPrescribed zinc for treatment	1.0^a^	Prescribed zinc at health facility	
ȃNo zinc prescription for treatment	4.18	Not prescribed zinc at health facility	
No antibiotic for dysentery (among cases with dysentery)			[5, 13]
ȃReceived or prescribed appropriate antibiotic for dysentery	1.0^a^	Administered or prescribed antibiotics for dysentery at health facility (including ceftriaxone, ciprofloxacin, pivmecillinam)	
ȃNo antibiotic for dysentery	1.21	Not prescribed antibiotics for dysentery (ceftriaxone, ciprofloxacin, or pivmecillinam) at health facility	

Abbreviations: GEMS, Global Enteric Multicenter Study; HAZ, height-for-age *z* score; ORS, oral rehydration salts; Ref, reference; VIDA, Vaccine Impact on Diarrhea in Africa; WAZ, weight-for-age *z* score; WHZ, weight-for-height *z* score.

Indicates risk factor reference group for prevalence calculations (not having risk factor) and theoretical minimal risk exposure level (TMREL) for population-attributable fraction and decomposition analysis.

### Statistical Methods

For these analyses, we estimated the annual age- and site-specific number of diarrhea-associated deaths, defined as death within 14 days of study enrollment with a diarrheal episode. The proportion of enrolled cases with a diarrhea-associated death in each population-based age and site group for 1 year was multiplied by the total number of children seeking care for MSD at the study facility during the study period and divided by the *r* value to estimate the age- and site-specific total diarrhea deaths. Standardized mortality rates were calculated for site-specific and overall study mortality rates (see Supplementary Material for more detail). The absolute change in diarrhea mortality between GEMS and VIDA studies was calculated as the difference in standardized mortality rates.

Nine risk factors and interventions determined to be causally related to diarrhea incidence or mortality in the Global Burden of Disease (GBD) 2017 [2, 4] or Lives Saved Tool (LiST) [5] and available from GEMS and VIDA data were included in this analysis. These include health risk factors (stunting [4, 10], underweight [4, 10], wasting [4, 10]), household risk factors (unsafe sanitation [4, 9] and unsafe water [4, 9]), and interventions (oral rehydration salts [ORS] for diarrhea [11], zinc for diarrhea [5, 12], antibiotics for diarrhea with dysentery [5, 13], fully vaccinated against rotavirus [8]). We calculated the age-, site-, and study-specific prevalence of each risk factor from either the cases (for treatment factors) or controls (for health, household, and vaccination factors) and assumed the prevalence to be representative of the risk factor coverage in the overall study catchment area. The prevalence of health and household risk factors, as well as rotavirus vaccination, was taken from controls. Coverage with ORS and zinc for treatment of diarrhea was taken from the proportion of cases at enrollment. The proportion prescribed or given antibiotics is among children with diarrhea and dysentery. In order to present all interventions as risk factors, variables are expressed from the point of view that would result in an absence of providing a benefit (eg, not fully vaccinated or unimproved sanitation). Risk factor definitions and prevalence calculations are described in Table 1.

We used a counterfactual framework to calculate the population-attributable fraction (PAF) for each risk factor. The counterfactual level used was the level of exposure that presents lowest risk of diarrhea mortality, also known as the theoretical minimal risk exposure level (TMREL). The calculation includes population-level exposure to each risk factor, the relative risk for diarrhea given exposure to that factor, estimated population-level annual diarrhea mortality rate, and TMREL [15]. The relative risk for diarrhea mortality associated with risk factors was taken from previously published modeling analyses [4, 5, 8–11, 13, 16]. Relative risks as well as risk factor categories are described in Table 1. PAFs were estimated independently for each risk factor. As a result, the sum of risk factor PAFs is not equal to 100% in a given population, representing multiple ways to prevent a death from diarrhea. Where data are limited for the causal association between diarrhea mortality and risk factor, estimates for diarrhea morbidity were used, notably unsafe water and sanitation.

We assessed the effect of changes for each risk factor among children under 5 years on diarrhea mortality utilizing a counterfactual definition of risk factor burden, such that diarrhea mortality rate due to each risk factor was equivalent to the reduction expected given complete absence of that risk factor. We performed a decomposition of the effects of the change in risk factors between GEMS and VIDA on the diarrhea mortality rate for each site and age group while accounting for independent effects of population growth, population aging, and underlying mortality rate (see more details in the Supplementary Material) [17].

Uncertainty in all calculated estimates was estimated using bootstrapping. A total of 5000 draws with replacement is reflected using the 2.5th and 97.5th percentiles of the simulated draws from the posterior distribution and labeled as 95% confidence intervals (CIs).

This study was approved by the ethical review committees at the University of Maryland, Baltimore (HP-00062472); the Centers for Disease Control and Prevention (CDC) (reliance agreement 6729); The Gambia Government/Medical Research Council/Gambia at the London School of Hygiene and Tropical Medicine (1409); the Comité d’Ethique de la Faculté de Médecine, de Pharmacie, et d’Odonto-Stomatologie, Bamako, Mali (no number); and the Kenya Medical Research Institute Scientific and Ethics Review Unit in Siaya County, Kenya (SSE 2996). Informed, written consent was obtained from all participants prior to initiation of study procedures.

## RESULTS

### Diarrhea Mortality

An estimated 182.3 diarrhea deaths per 100 000 children (95% CI: 136.7, 231.3 deaths) under 5 years of age occurred annually in 3 GEMS country study catchment areas during the study period. The annual diarrhea mortality declined to an estimated 63.2 deaths per 100 000 children (95% CI: 39.2, 90.8 deaths) during the VIDA study period.

During VIDA, the highest rate of diarrhea mortality was seen in The Gambia (98.4 per 100 000; 95% CI: 49.9, 156.3), followed by Kenya (45.8 per 100 000; 95% CI: 17.0, 80.0), whereas the lowest rates were observed in Mali (26.3 per 100 000; 95% CI: 6.2, 54.8). Overall, there was a 65.3% (95% CI: −80.0%, −45.0%) relative decline in diarrhea-associated mortality between GEMS and VIDA. The largest relative decline in diarrhea mortality between GEMS and VIDA was seen in Kenya (85.9%; 95% CI: −95.1%, −71.5%) followed by Mali (78.0%; 95% CI: −96.0%, −36.3%) and The Gambia (32.2%; 95% CI: −69.1%, −39.6%) (Table 2).

**Table 2.** Diarrhea-Associated Deaths, Mortality by Country per 100 000 Person-Years

	VIDA Estimated Diarrhea Deaths per Year^a^	VIDA Population Size	VIDA Mortality (95% CI)	Absolute Change (95% CI)	Relative Change (95% CI)
Basse and Bansang,^b^ The Gambia	45.2	45 899	98.4 (49.9, 156.3)	−46.8 (−135.5, 38.0)	−32.2% (−69.1%, 39.6%)
Siaya County, Kenya	13.7	29 835	45.8 (17.0, 80.0)	−279.1 (−407.3, −158.1)	−85.9% (−95.1%, −71.5%)
Bamako, Mali	7.8	29 592	26.3 (6.2, 54.8)	−93.0 (−169.3, −23.2)	−78.0% (−96.0%, −36.3%)
Total	66.6	105 325	63.2 (39.2, 90.8)	−119.1 (−175.3, −66.1)	−65.3% (−80.0%, −45.0%)

Abbreviations: CI, confidence interval; VIDA, Vaccine Impact on Diarrhea in Africa.

Estimated diarrhea deaths is the sum of age-stratified mortality rates shown in Supplementary Table 1.

Bansang community included in VIDA only.

### Risk Factors

**Figure 1.**
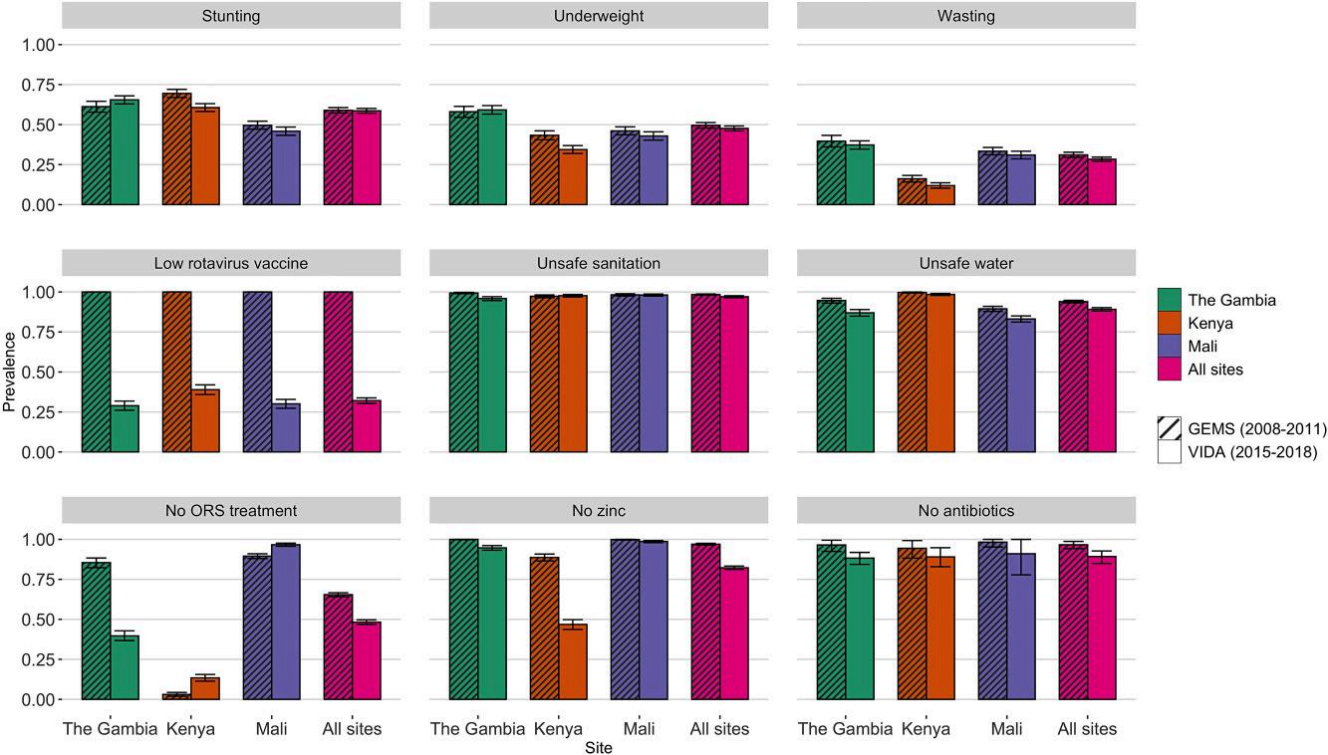
Prevalence of risk factors for diarrhea mortality by site and study. The population differed for prevalence calculation of prevention and treatment risk factors: prevalence of stunting, underweight, wasting, rotavirus vaccine, sanitation, and water among controls; prevalence of ORS treatment, zinc among cases; prevalence of antibiotics among cases with dysentery. Abbreviations: GEMS, Global Enteric Multicenter Study; ORS, oral rehydration salts; VIDA, Vaccine Impact on Diarrhea in Africa.

Prevalences of diarrhea risk factors in GEMS and VIDA are shown in Figure 1, and the absolute change between the 2 studies is presented in Supplementary Table 2. There were modest differences in prevalence of risk factors when assessing all sites combined, with a few exceptions. During GEMS, the rotavirus vaccine had not been used in any of the sites. In contrast, approximately 48% (95% CI: 47%, 50%) of children in VIDA were not fully vaccinated against rotavirus. Fewer children failed to receive ORS and zinc for treatment of their diarrhea in VIDA compared with GEMS, representing an increase in the proportion of children with diarrhea receiving these interventions. However, the trend varied by site. The majority of children with dysentery were not prescribed or administered an appropriate antibiotic in either GEMS and VIDA, but there was a consistent decline in this risk factor exposure across all sites, translating to an increase in the number of children with dysentery prescribed or administered an appropriate antibiotic.

**Figure 2.**
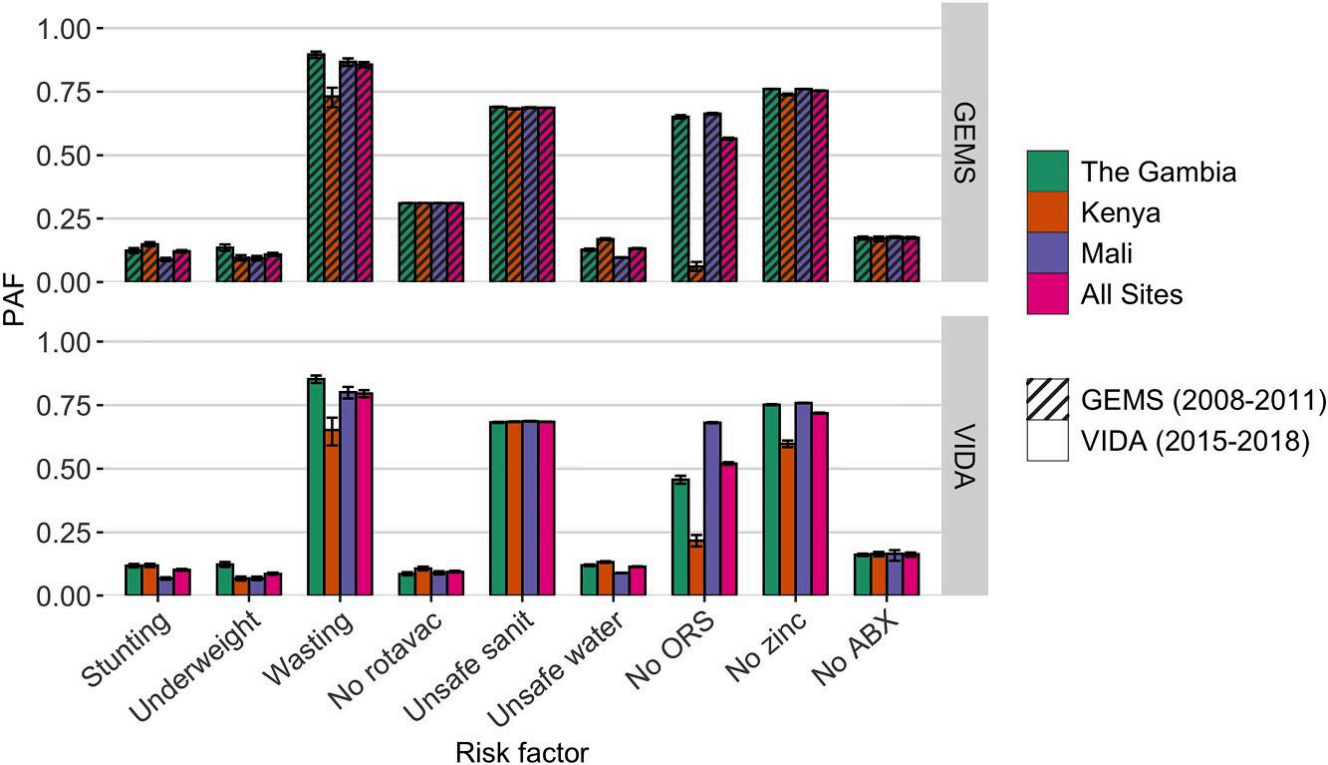
Population-attributable fractions for diarrheal risk factors for children under 5 across study sites and studies. Using the counterfactual risk framework, attribution of risk factors can overlap; 1 single risk may be sufficient but is not necessary to cause a diarrhea death. Abbreviations: ABX, antibiotics for dysentery; GEMS, Global Enteric Multicenter Study; ORS, oral rehydration salts; PAF, population-attributable fraction; rotavac, rotavirus vaccine; sanit, sanitation; VIDA, Vaccine Impact on Diarrhea in Africa.

Collectively, the risk factors in this analysis accounted for nearly all of the diarrhea deaths in the study catchment and during the VIDA study period (Figure 2; Supplementary Table 3). Wasting was attributed to 80% (95% CI: 78%, 81%) of diarrhea-associated deaths. A high proportion of diarrhea deaths during the VIDA period could be attributed to the low coverage of recommended treatment for diarrhea; 52% (95% CI: 52%, 53%) were attributed to a low prevalence of ORS treatment, 72% (95% CI: 72%, 72%) were attributable to a low prevalence of zinc treatment, and 16% (95% CI: 15%, 17%) are attributed to a low prevalence of antibiotic treatment for dysentery. The attributable fraction varied by site, however, showing that 68% (95% CI: 68%, 68%) of deaths in Mali and 22% (95% CI: 19%, 24%) in Kenya were attributed to failure to provide ORS for diarrhea. Full coverage of safe sanitation (indoor flush toilet connected to a sewer) and safe water (piped indoors) could have prevented 68% (95% CI: 68%, 69%) and 11% (95%: 11%, 12%), respectively, of diarrhea-associated deaths across all sites. Consistently across sites, preventing poor nutrition, including stunting and underweight, could have independently reduced diarrhea mortality by 10% (95% CI: 10%, 11%) and 9% (95% CI: 8%, 9%), respectively. Full use of the rotavirus vaccine could have averted 9% (95% CI: 9%, 10%) of deaths.

### Change in Mortality

**Figure 3.**
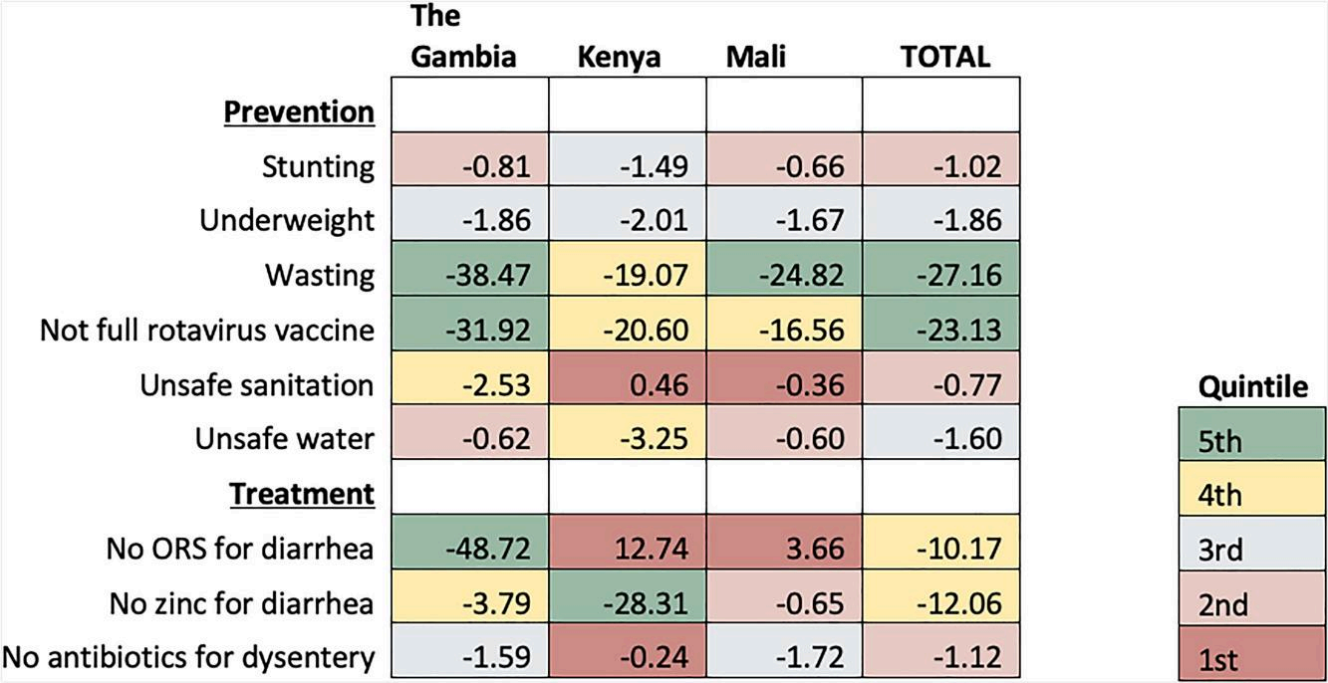
Percentage change in diarrhea mortality attributable to change in risk factors between GEMS and VIDA studies by country. Data are the percentage change in diarrhea mortality between GEMS and VIDA attributable to change in prevalence of each risk factor. The cell colors represent the quintile of the attributable change in mortality due to each risk factor among all countries, with green (5th quintile) having the largest impact on the decline of diarrhea mortality and red (1st quintile) having the least. A negative value indicates the percentage reduction in diarrhea mortality rate, a positive number indicates a reduction in diarrhea mortality was not attributed to a change in the risk factor. Abbreviations: GEMS, Global Enteric Multicenter Study; ORS, oral rehydration salts; VIDA, Vaccine Impact on Diarrhea in Africa.

The decomposition analysis of the percentage change in diarrhea mortality between the GEMS and VIDA study periods attributed to the change in diarrhea risk factors is shown in Figure 3, confidence intervals are also presented in Supplemental Table 4. In The Gambia, components of diarrhea treatment, improved nutritional status, and rotavirus vaccine introduction, all contributing to the decline in diarrhea mortality, accounted for some of the largest declines seen across all sites. A mortality rate decline of 48.7% (95% CI: −79.8%, −32.8%) was attributed to the expanded use of ORS for diarrhea and a 3.8% (95% CI: −6.0%, −2.5%) decline was attributed to the expanded use of zinc for diarrhea treatment. The second leading driver in diarrhea mortality decline in The Gambia was improvement in nutritional status, led by wasting (38.5%; 95% CI: −75.4%, −16.9%). Implementation of rotavirus vaccine was linked to a substantial reduction in diarrhea mortality at all sites, which was highest in The Gambia where it was linked to a 31.9% (95% CI: −50.9%, −22.4%) reduction.

Declines in Kenya were similar to those seen in The Gambia, with 1 major exception: the change in diarrhea mortality in Kenya could not be attributed to a change in ORS coverage or improvements in sanitation. The largest single factor associated with the decline in diarrhea mortality in Kenya was the use of zinc for diarrhea (28.3%; 95% CI: −36.1%, −23.3%). Similar to other sites, improvements in undernutrition risk factors as well as implementation of rotavirus vaccine were correlated with large declines in diarrhea mortality. A decrease in wasting prevalence was associated with a 19.1% (95% CI: −36.3%, −2.4%) decline in diarrhea mortality and expanded rotavirus vaccine coverage decreased diarrhea mortality in Kenya by 20.7% (95% CI: −25.8%, −17.3%).

Decreased mortality in Mali was mostly explained by improvements in nutritional status and rotavirus vaccine coverage. Moderate declines in the prevalence of wasting (2%) were linked to a 24.8% (95% CI: −41.7%, −12.6%) reduction in mortality rate. Consistent with other sites, implementation of rotavirus vaccine in Mali after the GEMS contributed to a 16.6% (95% CI: −23.3%, −13.6%) decline in diarrhea mortality. Administering appropriate antibiotics for dysentery reduced overall diarrhea mortality by 1.7% (95% CI: −6.3%, −.11%) in Mali.

## DISCUSSION

GEMS and VIDA study sites experienced an encouraging decline in diarrhea mortality between the 2008–2011 and 2015–2018 study periods. Our results suggest that reductions in the prevalence of wasting and rotavirus vaccine coverage were the leading drivers in this decline in diarrhea mortality in the 3 study sites, although leading contributing factors did vary between sites. These findings are derived from carefully conducted studies in which the mortality and all risk factors were directly measured in the same participants, complementing previous work using survey and modeled estimates.

Our results indicate that improvement in wasting prevalence was the largest contributor to the decline in diarrhea mortality between GEMS and VIDA across all study sites. Wasting is an important predictor of mortality during and after a diarrheal episode, acting as both a risk factor and consequence of MSD [18, 19]. As such, continued efforts to improve childhood nutritional status are warranted. A number of promising studies are underway testing the effect of various nutritional interventions that, if successful, could have a downstream effect of reducing diarrhea mortality.

The implementation of rotavirus vaccine between the GEMS and VIDA studies reduced diarrhea mortality by 23%, on average, at study sites. Our estimate is considerably lower than the estimated 36% reduction in acute gastroenteritis–related deaths after implementation of rotavirus vaccination noted in a recent meta-analysis, although this analysis did not determine that this reduction was specifically attributable to rotavirus vaccine [20]. While rotavirus vaccine introduction is a celebrated achievement, additional attention is needed to sustain and even improve this trend. First, the effect of rotavirus vaccine is markedly lower among children in low- compared with high-income countries [21]. Efficacy of the vaccines against severe rotavirus diarrhea is 90.6% in the Millennium Development Goal's Developed Region and only 46.1% in sub-Saharan Africa [22]. Despite significant efforts, researchers are not able to sufficiently explain this disparity. Second, many sub-Saharan countries are entering a critical period for rotavirus vaccine uptake as they transition from being eligible for Gavi-The Vaccine Alliance support.

Two recent publications include analyses assessing the effect of risk factors and interventions on global changes in childhood diarrhea mortality in recent decades. One, from the Global Burden of Disease (GBD) Consortium, modeled estimates for diarrhea and risk factor coverage and used decomposition methods, the method used in this analysis [4]. Another publication from Black et al [5] used national demographic survey data and vital registries to estimate diarrhea-associated mortality and risk factor coverage with the Lives Saved Tool (LiST). Country site-specific estimates of diarrhea-associated mortality rates differ considerably between the 3 analyses. The diarrhea mortality rate in Kenya and Mali from VIDA was 2–10 times lower than the national GBD estimates of 2017 and 2019, and both estimates were an order of magnitude lower than those estimated by Black and colleagues [4, 5, 23]. However, our mortality rate estimate for The Gambia site is comparable to the GBD national estimate. This discrepancy likely reflects differences in the study population compared with the countries as a whole as well as analytic differences. The GBD analysis points to improvements in unsafe sanitation and wasting as the 2 factors with the largest attribution globally, also listing wasting as the leading factor in Mali and Kenya. The LiST analysis, on the other hand, attributed the change in ORS for diarrhea and low vitamin A coverage as the key contributors for the decline in global diarrhea mortality. Despite including rotavirus vaccine in analysis, neither GBD nor LiST results suggest that it was a leading contributor in diarrhea mortality reduction globally or at the 3 selected study sites. Differences between these 3 studies could also be related to the estimation of risk factor coverage. Risk factor prevalence for our analysis is calculated from GEMS and VIDA participants who are representative of a specific community or region rather than the countries as a whole, the target of GBD and LiST analyses.

This study has several important limitations. First, these results are dependent on data from the GEMS and VIDA study populations and so may not represent the countries as a whole. The requirements that study sites be in close proximity to a laboratory and participants be seeking care for MSD limit the generalizability of the prevalence estimates to only similar communities. Second, there were slight changes between the GEMS and VIDA study. Notably, The Gambia added an additional community in the VIDA study that was not included in GEMS; thus, some of the change seen in The Gambia may be due to changes over time, but also changes in the study catchment area. However, the decomposition analysis does account for changes in population size, age distribution, and underlying mortality. Finally, this study was not well placed to examine the role of breastfeeding due to the enrollment strategy and information about vitamin A administration was not captured.

In conclusion, the GEMS and VIDA country sites—The Gambia, Kenya, and Mali—demonstrated exceptional reduction in diarrhea mortality over the last decade. While risk factor impact varied between sites, improvement in wasting and the implementation of rotavirus vaccine contributed to the decline in diarrhea mortality at all sites. Site-specific differences highlight an opportunity for implementation science in collaboration with policymakers to improve equitable coverage of these interventions globally, which will be required to further the declining trend of childhood diarrhea mortality.

## Supplementary Data

Supplementary materials are available at *Clinical Infectious Diseases* online. Consisting of data provided by the authors to benefit the reader, the posted materials are not copyedited and are the sole responsibility of the authors, so questions or comments should be addressed to the corresponding author.

## Supplementary Material

ciad015_Supplementary_Data

## Notes

***Acknowledgments*.** The authors express their deep gratitude to the families who participated in these studies, the clinical and field staff for their exceptional hard work and dedication, and to the physicians, administration, and health officials at every site who generously provided facilities and support for the conduct of the study. They are grateful to Catherine Johnson, Chris Focht, and Nora Watson at the Emmes Company, LLC, for expert data management and reporting. Special thanks go to Carl Kirkwood, Duncan Steele, and Anita Zaidi at the Bill & Melinda Gates Foundation for helpful oversight; Kathy Neuzil for thoughtful suggestions; and to the following members of our International Scientific Advisory Committee for providing insightful comments and guidance: Janet Wittes (Chair), George Armah, John Clemens, Christopher Duggan, Stephane Helleringer, Ali Mokdad, James Nataro, and Halvor Sommerfelt.

***Financial support*.** This work was supported by the Bill & Melinda Gates Foundation grants OPP1111236 and OPP1116751.

***Supplement sponsorship*.** This supplement was sponsored by the Bill & Melinda Gates Foundation. This study is based on research funded in part by grants from the Bill & Melinda Gates Foundation (OPP1111236/OPP1116751).
