# Supplementary material for: Drivers of Decline in Diarrhea Mortality Between GEMS and VIDA Studies
Source: Clin Infect Dis. 2023 Apr 19;76(Suppl 1):S58–65. doi: 10.1093/cid/ciad015 (PMC10116520; doi:10.1093/cid/ciad015)
Supplement: ciad015_Supplementary_Data [file ciad015_supplementary_data.docx]

**Supplemental: Exploring water, sanitation, and animal risk factors associated with enteric pathogen detection: Findings from the Vaccine Impact on Diarrhea in Africa (VIDA) Study, 2015 ⎯2018**

**Mortality calculations**

For these analyses, we estimated the annual age and site-specific number of diarrhea-associated deaths, defined as death within 14 days of study enrollment with a diarrheal episode. The proportion of enrolled cases with a diarrhea-associated death in each age and site group was multiplied by the total number of children seeking care for MSD at the study facility during the study period and divided by the product of the *r*-value and 3 years of enrollment to estimate the age and site specific total estimated diarrhea deaths. Diarrhea mortality for the study areas was calculated by dividing the total diarrhea deaths (the sum of estimated diarrhea deaths across three age strata) by the site population size (median of all DSS census rounds for the study period). The overall study mortality rate was calculate by dividing the total estimated diarrhea deaths (the sum of estimated deaths across age and site groups) divided by the total population (summed across sites).

**Decomposition analysis**

The decomposition analysis is based on methods developed by Das Gupta[24] and applied by Institute of Health Metrics and Evaluation in Global Burden of Disease analyses[25] to isolate drivers of burden changes by averaging across all contributing factors. The age (*a*), country site (*c*), and study (*s*) specific diarrhea mortality burden (R) can be determined using Das Gupta methods as product of the four components:

$$R_{acs}=(A_{acs}*B_{acs}*C_{acs}*D_{acs})$$

Where (A) population age structure (proportion of children in each specific age group), (B) the overall population size of children under 5, (C) underlying diarrhea mortality rate not attributed to risk factor (mortality rate *[1-PAF]), and (D) the inverse of the underlying rate reflecting risk factor exposure (1/[1- PAF]). The total rates for GEMS and VIDA as R_GEMS_ =ABCD and R_VIDA_=abcd. The contribution of each component to the total change in diarrhea mortality was determined by comparing change in risk factor prevalence between GEMS and VIDA while holding all other factors constant. The effect of a specific risk factor on the change in diarrhea mortality (Q) between studies is calculated as the average of all four factors:

$$Q_{D}=\left( d-D_{acs} \right)*(\frac{A_{acs}B_{acs}C_{acs}+ a_{acs}b_{acs}c_{acs}}{4}+ \frac{A_{acs}b_{acs}c_{acs}+A_{acs}B_{acs}c_{acs}+A_{acs}b_{acs}C_{acs}+a_{acs}B_{acs}c_{acs}+a_{acs}B_{acs}C_{acs}+a_{acs}b_{acs}C_{acs}}{12}$$

The risk factor effect (Q) divided by the diarrhea mortality in GEMS represents the percent change in diarrhea mortality attributable to change in risk factor prevalence.

**Supplementary Table 1**. Calculation of diarrhea-associated deaths by study, site, and age group.

|  |  | **GEMS** | | | | |  | **VIDA** | | | | |
| --- | --- | --- | --- | --- | --- | --- | --- | --- | --- | --- | --- | --- |
|  | **Diarrhea case deaths/ total cases** | **Total seeking care for MSD at Sentinel Facility** | **Proportion seeking care^1^** | **Average annual population deaths** | **Population size** | **Annual mortality rate per 10000** | **Diarrhea case deaths/ total cases** | **Total seeking care for MSD at Sentinel Facility** | **Proportion seeking care^1^** | **Average annual population deaths** | **Population size** | **Annual mortality rate per 10000** |
| **Basse & Bansang**^2^**,**  **The Gambia** |  |  |  |  |  |  |  |  |  |  |  |  |
| **0-11m** | 8/400 | 797 | 0.35 | 15.18 | 5708 | 26.6 | 6/540 | 1175 | 0.26 | 16.97 | 6068 | 27.96 |
| **12—23m** | 7/455 | 927 | 0.26 | 18.28 | 6230 | 29.35 | 6/618 | 1832 | 0.29 | 20.17 | 8546 | 23.6 |
| **24-59m** | 3/174 | 335 | 0.22 | 8.75 | 17139 | 5.11 | 2/520 | 1073 | 0.17 | 8.02 | 31285 | 2.56 |
| **Nyanza Province, Kenya** |  |  |  |  |  |  |  |  |  |  |  |  |
| **0-11m** | 15/673 | 994 | 0.2 | 36.92 | 3159 | 116.89 | 4/586 | 683 | 0.25 | 6.17 | 6821 | 9.05 |
| **12—23m** | 8/410 | 599 | 0.19 | 20.5 | 4746 | 43.2 | 4/528 | 651 | 0.22 | 7.51 | 5528 | 13.58 |
| **24-59m** | 5/393 | 481 | 0.16 | 12.75 | 13698 | 9.31 | 0/440 | 520 | 0.15 | 0 | 17486 | 0 |
| **Bamako, Mali** |  |  |  |  |  |  |  |  |  |  |  |  |
| **0-11m** | 9/727 | 1654 | 0.22 | 31.02 | 6448 | 48.11 | 3/595 | 653 | 0.19 | 5.83 | 5956 | 9.79 |
| **12—23m** | 2/682 | 1194 | 0.17 | 6.87 | 6803 | 10.09 | 0/552 | 619 | 0.15 | 0 | 6003 | 0 |
| **24-59m** | 0/624 | 763 | 0.09 | 0 | 18517 | 0 | 1/461 | 492 | 0.18 | 1.95 | 17633 | 1.1 |

^1^Proportion of children under 5 interviewed in community with diarrhea who sought care at study health facility (r-value)

^2^Bansang community included in VIDA only

Estimated number of deaths shown in Table 1 was calculated by summing together estimated deaths for each age group.

**Supplementary Table 2**: Prevalence of risk factor during VIDA and percent change of exposure

| **Site** | **The Gambia** | | **Kenya** | | **Mali** | | **All Sites** | |
| --- | --- | --- | --- | --- | --- | --- | --- | --- |
|  | **VIDA**  **prevalence (95%: CI)** | **Absolute change** | **VIDA**  **% (95%: CI)** | **Absolute change** | **VIDA**  **% (95%: CI)** | **Absolute change** | **VIDA**  **% (95%: CI)** | **Absolute change** |
| **Stunting** | 0.65 (0.63, 0.68) | 0.04 (0.00, 0.08) | 0.61 (0.58, 0.63) | -0.09 (-0.12, -0.05) | 0.46 (0.43, 0.48) | -0.04 (-0.07, 0.00) | 0.59 (0.57, 0.60) | 0 (-0.03, 0.02) |
| **Underweight** | 0.59 (0.57, 0.62) | 0.01 (-0.03, 0.05) | 0.34 (0.32, 0.37) | -0.09 (-0.13, -0.05) | 0.43 (0.40, 0.45) | -0.03 (-0.07, 0.00) | 0.48 (0.46, 0.49) | -0.02 (-0.04, 0.00) |
| **Wasting** | 0.37 (0.35, 0.4) | -0.02 (-0.07, 0.02) | 0.12 (0.10, 0.14) | -0.04 (-0.07, -0.02) | 0.31 (0.29, 0.33) | -0.02 (-0.06, 0.01) | 0.28 (0.27, 0.30) | -0.03 (-0.05, -0.01) |
| **No full Rotavirus vaccine** | 0.4 (0.37, 0.43) | -0.46 (-0.50, -0.42) | 0.13 (0.11, 0.16) | 0.10 (0.08, 0.13) | 0.97 (0.96, 0.98) | 0.07 (0.05, 0.09) | 0.48 (0.47, 0.50) | -0.17 (-0.19, -0.15) |
| **Unsafe sanitation** | 0.95 (0.93, 0.96) | -0.05 (-0.07, -0.04) | 0.47 (0.44, 0.50) | -0.42 (-0.46, -0.38) | 0.99 (0.98, 0.99) | -0.01 (-0.02, -0.01) | 0.82 (0.81, 0.83) | -0.15 (-0.16, -0.13) |
| **Unsafe water** | 0.88 (0.84, 0.92) | -0.08 (-0.13, -0.03) | 0.89 (0.83, 0.95) | -0.05 (-0.13, 0.03) | 0.91 (0.78, 1.00) | -0.07 (-0.21, 0.02) | 0.89 (0.85, 0.93) | -0.07 (-0.12, -0.03) |
| **No ORS for diarrhea** | 0.29 (0.26, 0.32) | -0.71 (-0.74, -0.68) | 0.39 (0.36, 0.42) | -0.61 (-0.64, -0.58) | 0.30 (0.27, 0.33) | -0.70 (-0.73, -0.67) | 0.32 (0.30, 0.34) | -0.68 (-0.7, -0.66) |
| **No zinc for diarrhea** | 0.96 (0.95, 0.97) | -0.03 (-0.05, -0.02) | 0.98 (0.97, 0.98) | 0 (-0.01, 0.02) | 0.98 (0.97, 0.99) | 0 (-0.01, 0.01) | 0.97 (0.96, 0.98) | -0.01 (-0.02, -0.01) |
| **No antibiotics for dysentery** | 0.87 (0.85, 0.89) | -0.08 (-0.1, -0.05) | 0.98 (0.98, 0.99) | -0.01 (-0.02, -0.01) | 0.83 (0.81, 0.85) | -0.06 (-0.09, -0.04) | 0.89 (0.88, 0.90) | -0.05 (-0.06, -0.04) |

**Supplementary Table 3**: PAF of risk factor during by site and study

| **Site** | **The Gambia** | | **Kenya** | | **Mali** | | **All Sites** | |
| --- | --- | --- | --- | --- | --- | --- | --- | --- |
|  | **GEMS**  **PAF (95%: CI)** | **VIDA**  **PAF (95%: CI)** | **GEMS**  **PAF (95%: CI)** | **VIDA**  **PAF (95%: CI)** | **GEMS**  **PAF (95%: CI)** | **VIDA**  **PAF (95%: CI)** | **GEMS**  **PAF (95%: CI)** | **VIDA**  **PAF (95%: CI)** |
| **Stunting** | 0.12 (0.11, 0.13) | 0.12 (0.11, 0.13) | 0.15 (0.14, 0.16) | 0.12 (0.11, 0.13) | 0.09 (0.08, 0.1) | 0.07 (0.06, 0.07) | 0.12 (0.12, 0.13) | 0.1 (0.1, 0.11) |
| **Underweight** | 0.14 (0.12, 0.15) | 0.12 (0.11, 0.13) | 0.1 (0.09, 0.11) | 0.07 (0.06, 0.07) | 0.09 (0.09, 0.1) | 0.07 (0.06, 0.07) | 0.11 (0.1, 0.11) | 0.09 (0.08, 0.09) |
| **Wasting** | 0.9 (0.88, 0.91) | 0.85 (0.84, 0.87) | 0.73 (0.69, 0.77) | 0.65 (0.59, 0.7) | 0.87 (0.85, 0.88) | 0.8 (0.78, 0.82) | 0.86 (0.85, 0.87) | 0.8 (0.78, 0.81) |
| **Not full Rotavirus vaccine** | 0.31 (0.31, 0.31) | 0.09 (0.08, 0.09) | 0.31 (0.31, 0.31) | 0.11 (0.1, 0.11) | 0.31 (0.31, 0.31) | 0.09 (0.08, 0.1) | 0.31 (0.31, 0.31) | 0.09 (0.09, 0.1) |
| **Unsafe sanitation** | 0.69 (0.69, 0.69) | 0.68 (0.68, 0.68) | 0.68 (0.68, 0.68) | 0.68 (0.68, 0.69) | 0.69 (0.69, 0.69) | 0.69 (0.69, 0.69) | 0.69 (0.69, 0.69) | 0.68 (0.68, 0.69) |
| **Unsafe water** | 0.13 (0.12, 0.13) | 0.12 (0.12, 0.12) | 0.17 (0.16, 0.17) | 0.13 (0.13, 0.14) | 0.1 (0.1, 0.1) | 0.09 (0.09, 0.09) | 0.13 (0.13, 0.13) | 0.11 (0.11, 0.12) |
| **No ORS for diarrhea** | 0.65 (0.64, 0.66) | 0.46 (0.44, 0.47) | 0.06 (0.04, 0.08) | 0.22 (0.19, 0.24) | 0.66 (0.66, 0.67) | 0.68 (0.68, 0.68) | 0.57 (0.56, 0.57) | 0.52 (0.52, 0.53) |
| **No zinc for diarrhea** | 0.76 (0.76, 0.76) | 0.75 (0.75, 0.75) | 0.74 (0.73, 0.74) | 0.6 (0.58, 0.61) | 0.76 (0.76, 0.76) | 0.76 (0.76, 0.76) | 0.75 (0.75, 0.75) | 0.72 (0.72, 0.72) |
| **No antibiotics for dysentery** | 0.17 (0.17, 0.18) | 0.16 (0.16, 0.17) | 0.17 (0.16, 0.18) | 0.16 (0.16, 0.17) | 0.18 (0.17, 0.18) | 0.17 (0.14, 0.18) | 0.17 (0.17, 0.18) | 0.16 (0.15, 0.17) |

No child was vaccinated against rotavirus in GEMS

**Supplementary Table 4**: Percent change of diarrhea mortality between GEMS and VIDA attributable to risk factor

| **Site** | **The Gambia** | **Kenya** | **Mali** | **All Sites** |
| --- | --- | --- | --- | --- |
|  | **Percent change (95%: CI)** | **Percent change (95%: CI)** | **Percent change (95%: CI)** | **Percent change (95%: CI)** |
| **Stunting** | -0.81 (-2.39, 0.63) | -1.49 (-2.63, -0.34) | -0.66 (-1.43, 0.03) | -1.02 (-1.72, -0.34) |
| **Underweight** | -1.86 (-4.75, 0.47) | -2.01 (-3.39, -0.62) | -1.67 (-2.96, -0.48) | -1.86 (-2.92, -0.84) |
| **Wasting** | -38.47 (-75.41, -16.93) | -19.07 (-36.29, -2.37) | -24.82 (-41.67, -13.61) | -27.16 (-39.33, -16.79) |
| **Not full Rotavirus vaccine** | -31.93 (-50.86, -22.36) | -20.6 (-25.77, -17.25) | -16.56 (-23.3, -13.62) | -23.13 (-28.35, -19.44) |
| **Unsafe sanitation** | -2.53 (-4.15, -1.55) | 0.46 (-0.12, 1.08) | -0.36 (-0.92, 0.12) | -0.77 (-1.29, -0.30) |
| **Unsafe water** | -0.62 (-1.53, 0.11) | -3.25 (-4.04, -2.62) | -0.60 (-0.89, -0.40) | -1.60 (-2.12, -1.16) |
| **No ORS for diarrhea** | -48.72 (-79.81, -32.84) | 12.74 (9.58, 16.49) | 3.66 (2.45, 5.41) | -10.17 (-17.65, -4.25) |
| **No zinc for diarrhea** | -3.79 (-6.02, -2.53) | -28.31 (-36.08, -23.33) | -0.65 (-1.16, -0.27) | -12.06 (-16.03, -8.86) |
| **No antibiotics for dysentery** | -1.59 (-3.12, -0.45) | -0.24 (-1.74, 1.54) | -1.72 (-6.34, 0.11) | -1.12 (-2.66, 0.01) |
